# Validation of reference genes for gene expression analysis in fruit development of *Vaccinium bracteatum* Thunb. using quantitative real-time PCR

**DOI:** 10.1038/s41598-022-20864-7

**Published:** 2022-10-09

**Authors:** Feng He, Liangxian Gui, Yan Zhang, Bo Zhu, Xiaoping Zhang, Min Shen, Fengying Wan, Lu Yang, Jiaxin Xiao

**Affiliations:** College of Life Sciences, Anhui Normal University, Wuhu, 241000 China

**Keywords:** Genetics, Molecular biology, Plant sciences

## Abstract

*Vaccinium bracteatum* Thunb. (VBT) is widely distributed in the mountainous areas in eastern and southern China. VBT leaves have great medical value and can be used to stain rice to produce “Wumifan”. Its fruits also contain rich nutrients. However, there has been limited attention to exploring the molecular content of VBT. Previously, we performed RNA-seq on three typical VBT fruits that were at various stages of ripening, although a reliable reference gene was lost in validation.In this study, we selected ten candidate reference genes based on previous studies and transcriptomics analyses. Subsequently, these genes were evaluated using a combination of methods, including geNorm, NormFinder, and Bestkeeper, with a comprehensive ranking assessment. As a result, we found that the actin2, NADH, and ADK genes have high reliability for analysing the expression levels of genes involved in fruit development. Furthermore, the transcript levels of 15 DEGs from transcriptomic analysis were assessed using NADH as a reference gene, and RT-qPCR data were highly consistent with the transcriptomic data. These results provide reliable reference genes for further studying gene expression, which will be beneficial for comprehensively exploring VBT.

## Introduction

*Vaccinium bracteatum* Thunb. (VBT), which belongs to the Ericaceous family and is also called wild blueberry, “Nanzhu”, and “Wufanzi”, is diffusely distributed in mountainous areas of eastern and southern China^1,2^. The plant is famous for producing “Wumifan”, as its leaves contain pigments that can be used to stain the rice. Although VBT fruit is like blueberries, its phenological period is different from that of blueberries. The flowering phase of VBT is June, while fruit ripen in November and December, and its fruit is smaller than blueberries. The species is also used extensively as an herbal medicine in China, the original description as an herbal medicine was in the Tang Dynasty, recorded by the book “Ben Cao Shi Yi”^3,4^. Recently, the species was demonstrated to confer prominent benefits for hypoglaemia, sedation and hypnosis, antineoplastic, antimicrobial and antioxidant activity^3–9^. Additionally, VBT contains high levels of nutrients, such as amino acids, minerals and unsaturated fatty acids^3,5,10^. Therefore, VBT is an important resource for the traditional diet as well as for its pharmacological effects, for which it has considerable prospects for development.

Recently, an increasing number of studies have focused on the herbal effects of VBT and its potential prospects in food production. Polysaccharides^11^ and flavonoids^12^ were reported as major components of VBT leaves. In addition, several reports focused on the formation of “Wumi”^2,6,13,14^. The findings showed that iridoid glycosides may be the precursor compound, among a profile of pigments, to bind to rice protein molecules^15^. These studies provided novel insights for understanding VBT and identified prospects for exploration of VBT products. However, most of these studies focused on chemical compounds^4,7,11,16,17^, and very few studies have concentrated on VBT’s genetic and biochemically active molecules, which may promote its application in improving the breeding of crops.

Since the leaves and fruit of VBT have great benefits to human health, the nutrient and key component synthetic pathways should be further elucidated. To discover the molecular mechanism of VBT in pigment synthesis, nutrient enrichment, and the production of other bioactive components, genomics and transcriptomics analyses offer effective methods. Transcritpomics analysis is needed to confirm the quantitative, real-time, reverse transcriptase polymerase chain reaction (RT–qPCR), which is always used to test gene expression levels in biological samples and tissues^18–20^. RT–qPCR was calculated based on reference genes, such as actin^21,22^, tubulin^22^, EF1^20,22^, GAPDH^21^, ubiquitin^23^, and others. Actin, GAPDH, tubulin, and ubiquitin are used commonly in analysing gene expression levels in plants^23–26^. However, there are no standards to use as reference genes in various species or tissues^24^. The best reference genes were generally confirmed by analytical tools, such as GeNorm, NormFinder, and BestKeeper^25^. However, the scores for reference genes evaluated sing these three methods are often not uniform. Therefore, researchers have selected genes that have high scores in GeNorm and NormFinder or used a combination of the three methods^24^. As a result, the best reference gene remains to be identified.

VBT is a unique species in China. Because of its high medicinal value and rich nutrient qualities, the molecular mechanisms involved in nutrient and active biochemical component synthesis in VBT should be better understood. Thus, our group sequenced the genome and transcriptome of VBT. However, validation of the differentially expressed genes in the transcriptome involved in various fruit- development stages using the *actin* gene as a normalizer was not stable and the results showed pronounced variability across samples of fruits at the same developmental stage. Moreover, the best reference gene in the development of fruits is not *actin* which, therefore, implies that it is not adaptable for validation of genes involved in fruit development. Consequently, we selected ten candidate reference genes from the VBT transcriptomic data that had similar gene expression levels in different fruit development stages. Additionally, we evaluated the ten candidates using GeNorm, NormFinder, and BestKeeper. The results will benefit the future analysis of gene expression in VBT.

## Methods and materials

### Sample collection, RNA extraction, and cDNA synthesis

Liangxian Gui collected the fruits from a blue berry orchard (Anhui Huiwang Agricultural Company, 30°51′N, 118°23′E) in Wuhu city, and permission were obtained from the person in charge. The fruits were collected by from a blue berry orchard. According to monitoring fruit ripeness, we went to the company at different times. Then, three green (S1), red (S2), and blue (S3) fruits were collected in 50 mL tubes, and three tubes were prepared for each fruit ripening stage. At the same time, they were placed in liquid nitrogen and later kept in an ultracold storage freezer for future study.

Total RNA of fruits from the three ripening stages was extracted using a mirVana miRNA Isolation kit (Ambion). The total RNA was later evaluated using gel electrophoresis, and integrity was assessed using Agilent Technologies (Santa Clara, CA, USA). Once the RNA samples were of good quality, cDNA was synthesized using a PrimeScript RT reagent kit with gDNA Eraser (Perfect Real Time, Takara, Japan) following the manufacturer’s instructions.

### Selection of candidate reference genes

According to a previous study and the transcriptomics data, ten candidate reference genes were selected. The genes consist of RP (50S ribosomal protein, CL31684Contig1), actin5 (actin related protein-5, CL33133Contig1), UBQ (Ubiquitin domain-containing protein, CL33177Contig1), actin1 (actin related protein-1,CL34455Contig1), actin4 (actin related protein-4, CL8799Contig1), UBE (Ubiquitin-conjugating enzyme, CL31240Contig1), actin3 (actin related protein-3, CL40530Contig1), actin2 (actin related protein-2, CL11169Contig1), NADH (NADH dehydrogenase, CL31981Contig1), and ADK (Adenylate kinase, CL27968Contig1) (Table S2). These genes had similar FPKM values in the three ripening stages according to the de novo transcriptomics analysis.

Primers for the candidate reference genes and DEGs were designed using Premier Primer 6 software based on the following criteria: primer length of ~ 18–22 bp, melting temperature 59–61 °C, GC content 40–60%, and amplicon length of 80–200 bp. Later, the primers were synthesized by Sangon Biotech Company (Shanghai, China) (Table S2).

### RT–qPCR and data analyses

RT–qPCR was performed using Tiangen SuperReal PreMix Plus (SYBR Green) kit (Tiangen, Beijing, China) on Bio-Rad CFX Manager (Bio-Rad, CA, USA) following the manufacturer’s instructions. When RT–qPCR was completed, the melting curve and cycle threshold (Ct) values were promptly exported to confirm primer specificity and expression levels of the reference gene, respectively. The stability of the candidate reference genes was evaluated using geNorm, NormFinder, and BestKeeper, and comprehensive rankings were assessed by RefFinder (http://blooge.cn/RefFinder/) based on the Ct values. The genes ACO, PG, and other DEGs were acquired from transcriptomics, and their primers were designed for RT–qPCR (Table S2). The expression levels of the genes were normalized to the transcript levels of the genes NADH, ADK, actin5, and NADH+ADK to confirm the reference genes. The DEGs were further analysed using the NADH, and the Pearson correlation value (R) between the RT–qPCR data and RNA-seq data was analysed using the formulas in Excel. Each RT–qPCR experiment was repeated at least three times.

We ensured that all methods were carried out in accordance with managements of the company and local regulations. For this study, only the fruits or leaves were collected, however, the plants are not destroyed.

## Results

### Prediction of candidate reference genes in VBT transcriptomics and primer design

**Figure 1:**
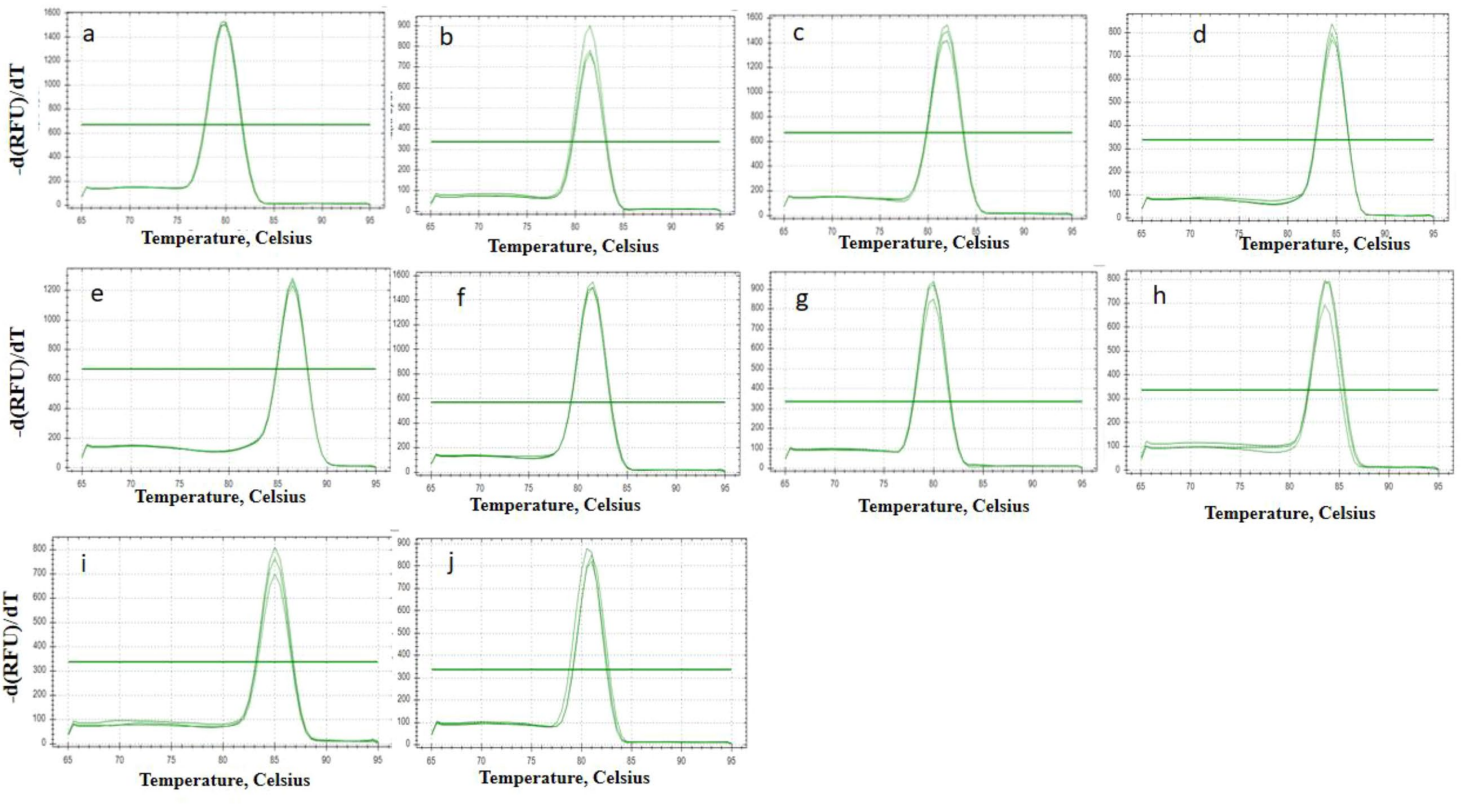
Melting curves of the reference genes based on RT-qPCR data. (**a**) actin1. (**b**). actin2. (**c**) actin3. (**d**) actin4. (**e**). actin5. (**f**) RP. (**g**) UBE. (**h**) UBQ. (**i**) NADH. (**j**) ADK. The melting curves for each gene was generated on Bio-Rad CFX Manager according to instructions.

The fruit transcriptome includes green fruits, red fruits and blue fruits, which represent the progressive stages of fruit ripeness. ^27^ According to previous studies and the transcriptomic analysis, we identified ten reference genes. There were five actin-related genes (actin-related protein 2/3 complex subunit 2A (actin 1), actin-7 (actin 2), actin-depolymerizing factor 2 (actin 3), actin-97 (actin 4), and actin-related protein 3 (actin 5)), 50S ribosomal protein L27 (RP), ubiquitin-conjugating enzyme E25 (UBE), ubiquitin domain-containing protein DSK2b (UBQ), NADH dehydrogenase (NADH), and adenylate kinase 4 (ADK), all of which have been widely used for gene validation in plants and fruits (Table S1). Simultaneously, RNA from the green, red, and blue fruits was extracted, and pure total RNA was quantified. Furthermore, the primers for RT–qPCR were designed (Table S2), and later these primers were confirmed using melting curves. The results showed that a single peak for each primer pair was present, which indicates exceptionally high primer specificity (Fig. 1).

### Expression levels of the candidate reference genes

**Figure 2:**
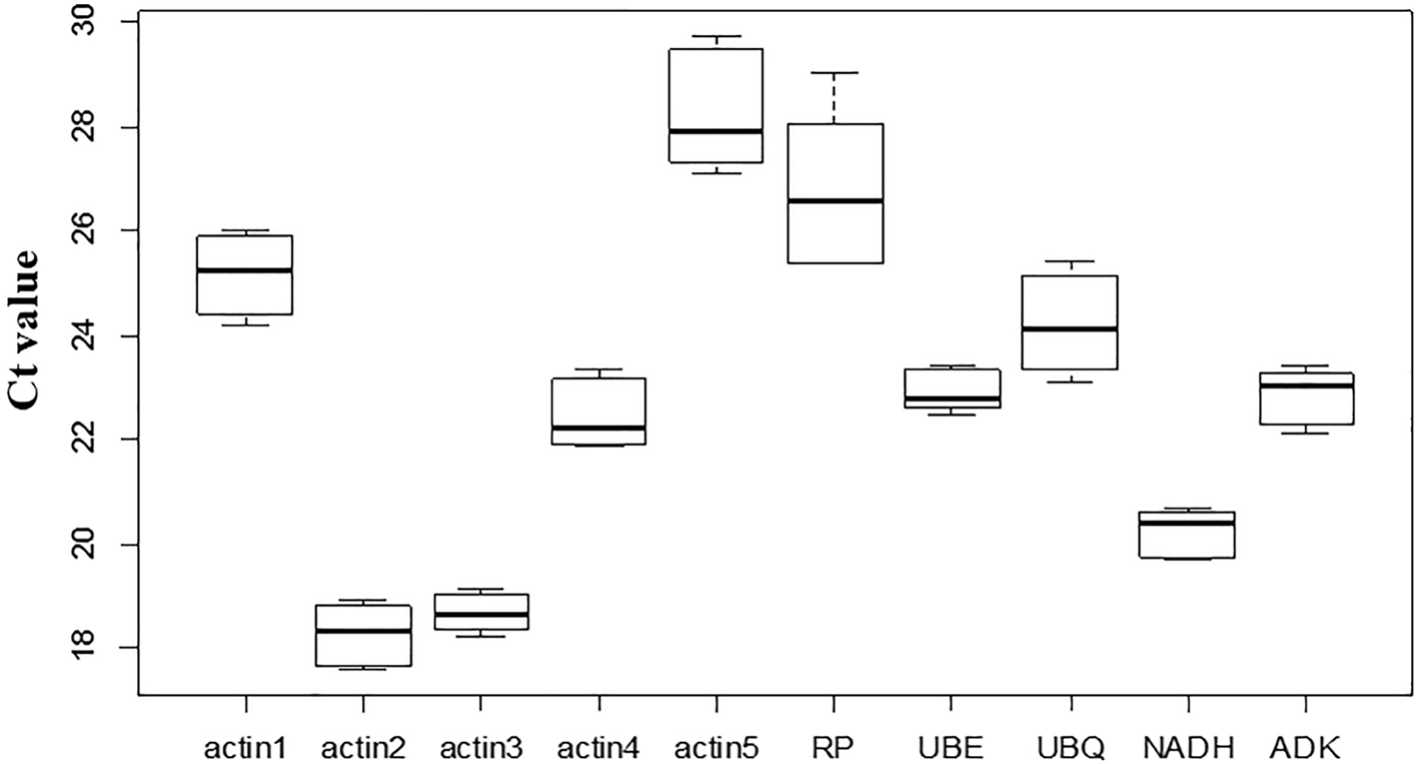
Ct values variance among three fruit development stage. RT-qPCR was performed using Bio-Rad CFX Manager, and Ct values of each reference genes in the samples (S1, S2 and S3) were acquired later. The picture were drawn by R, using these Ct values.

To evaluate the expression levels of the candidate reference genes, the cycle threshold (Ct) values of the ten candidate reference genes in green, red, and blue fruits were acquired with three replicates. The expression levels of these ten reference genes were dramatically varied, with Ct values ranging from 17.61 to 29.73 cycles. According to the Ct values, actin 2 expression levels in the three fruit samples were the highest, and actin 5 was the lowest (Fig. 2). In the green fruits, all the gene expression levels ranged from actin2 > actin3 > NADH > actin4 > UBE > ADK > UBQ > actin1 > RP > actin5 (Table S3). In thered and blue fruits, the arrangement changed in the order actin2 > actin3 > NADH > actin4 > ADK > UBE > UBQ > actin1 > RP > actin5 (Table S3). According to these data, we also found that the expression levels of *actin3*, *NADH*, *ADK*, and *UBE* had narrow variances across the three fruit development stages, which convincingly suggested that their expression levels were stable in the three fruits (Fig. 2). These data demonstrated that these genes could be used as reference genes.

### Expression stability of candidate reference genes

#### geNorm analysis

**Figure 3:**
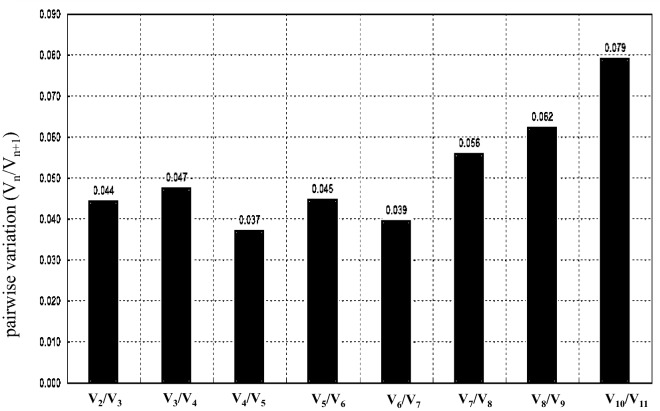
Pairwise variation (Vn/Vn + 1) values for three fruit ripen stage. Vn/Vn + 1 > 0.15 indicates another (n + 1) reference was required, while Vn/Vn + 1 ≤ 0.15 demontrates only n reference was enough.

The average expression stability (M value) of the ten genes was calculated by geNorm (version 3.5). The M values of all ten genes across green, red, and blue fruits were lower than the threshold of 1.5, which means that all the selected genes could be used as reference genes (Table 1). However, the M value of these genes showed a great variability; the M value of the RP gene was the highest, while those of the NADH and ADK genes were the smallest, suggesting that they had the highest expression stability (Table 1). These results showed that NADH and ADK are the best reference genes, followed by actin2. To further assess the optimal number of reference genes, pairwise variation (V_n_/V_n + 1_) was analysed using geNorm, and 0.15 was used as a cut-off value according to a previous study. The results showed that all the V_n_/V_n + 1_ values were less than 0.15. Moreover, the pairwise variation (V2/V3) value was significantly less than 0.15 (Fig. 3), suggesting that only two reference genes were necessary for the gene profile; thus, we surmised that no additional reference genes were required.

**Table 1:** Analysis of expression stability and ranking of candidate reference genes by geNorm, NormFinder and BestKeeper.

Ranking	geNorm		NormFinder		Bestkeeper			
	Genes	M value	Genes	Stability value	Genes	SD	Genes	CV%
1	NADH	0.096	actin1	0.03	actin3	0.28	UBE	1.37
2	ADK	0.096	actin4	0.098	UBE	0.31	actin3	1.5
3	actin2	0.133	actin2	0.139	NADH	0.35	NADH	1.74
4	actin3	0.168	ADK	0.261	ADK	0.42	ADK	1.85
5	UBE	0.181	UBQ	0.266	actin2	0.43	actin1	2.30
6	actin4	0.216	UBE	0.322	actin4	0.52	actin4	2.33
7	actin1	0.242	NADH	0.336	actin1	0.58	actin2	2.35
8	UBQ	0.297	actin3	0.350	UBQ	0.73	UBQ	3.01
9	actin5	0.359	actin5	0.464	actin5	0.89	actin5	3.15
10	RP	0.449	RP	0.785	RP	0.93	RP	4.09

#### NormFinder analysis

In addition to the geNorm analysis, NormFinder analysis is also a necessary method for confirming the best reference gene. The gene with the smallest S-value was the most suitable reference gene. According to the NormFinder analysis, the S-value of all the candidate genes was less than 1.0, which indicates that all the reference genes we evaluated could be used for RT–qPCR analysis. Of them, the S-value of the *actin1* gene (0.03) was the smallest and that of RP (0.785) was the largest (Table 1), which indicates that the actin1 gene is the most stable reference gene, and the results were not uniform with geNorm analysis.

#### BestKeeper analysis

Finally, BestKeeper was used to evaluate the stability of the reference genes through the standard deviation (SD) and the coefficient of variance (CV) of the Ct values. If the SD value of the Ct values were > 1, the genecould not be set as a reference gene. According to BestKeeper analysis, both Ct values of all the genes were smaller than 1.0, which indicates that all the genes we selected met the standard of BestKeeper. Among them, however, actin3, UBE, and NADH are the three genes with lowest SD values. Like the SD value, these three genes had smaller CV values (Table 1). However, UBQ, actin5, and RP both had higher SD values and CV values (Table 1). Thus, while these results demonstrate that all the ten genes we selected could be used as reference genes, the actin3, UBE, and NADH genes are the best choices overall.

#### Recommended comprehensive ranking

Since the analysis by the three methods is not uniform, we compared and ranked the reference genes by RefFinder, which is a comprehensive, web-based tool combined with geNorm, Normfiner, BestKeeper, and Ct values. The overall final ranking based on the ranking from each program is shown in Table 2. These results showed that *actin2*, ADK, *actin1*, and NADH were the four genes with smaller values, and the overall ranking values (*actin2*, 2.59; ADK, 2.83; *actin1*, 3.15; NADH, 3.20) were very close (Table 2). However, partial Ct values of *actin1* in red fruit were over 26, suggesting that its expression levels are slightly low and may not be suitable for RT–qPCR analysis of genes in all stages of fruit development. Considering all these results, we conclude that the three reference genes, *actin2*, ADK and NADH, are the best reference genes for RT–qPCR analysis across all stages of fruit development.

**Table 2:** Recommended comprehensive ranking order (Better–Good–Average).

Method	1	2	3	4	5	6	7	8	9	10
Delta CT	actin2	actin1	actin4	ADK	NADH	UBE	actin3	UBQ	actin5	RP
BestKeeper	actin3	UBE	NADH	ADK	actin2	actin4	actin1	UBQ	actin5	RP
Normfinder	actin1	actin4	actin2	ADK	UBQ	UBE	NADH	actin3	actin5	RP
Genorm	NADH/ADK		actin2	actin3	UBE	actin4	actin1	UBQ	actin5	RP
Recommended comprehensive ranking	actin2	ADK	actin1	NADH	actin4	actin3	UBE	UBQ	actin5	RP

#### Identification of reference genes using marker genes in fruit development

**Figure 4:**
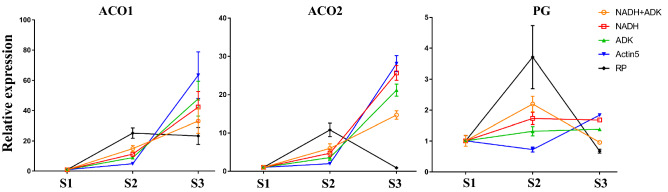
Evaluation of reference genes using the mark genes involved in fruit ripening. S1 indicates the green fruits, S2 indicates the red fruits, S3 indicates the blue fruits. Relative expression levels ot the three genes were normalized to reference genes NADH, ADK, NADH + ADK, RP, and *actin*5, respectively. Each RT-qPCR experiment was repeated at least three times. Labels for each reference genes as normalizer were shown in the picture. Pictures were drawn using GraghPad Prism 7.0.

Since ACO and PG genes are generally upregulated in fruit ripening programs, assessing their expression levels should be the best way to confirm the reliability of reference genes. Additionally, the ranking scores of the three best reference genes (*actin2*, ADK and NADH) is close, which means any one is suitable for gene expression analysis. To assess the reference gene, the expression levels of ACO1, ACO2, and PG were calculated using NADH, ADK, NADH + ADK, RP and actin5. We found that the transcript levels of ACO genes normalized by RP were not in accordance with their expression features (Fig. 4). Moreover, the transcript levels of PG normalized by actin5 in red fruit did not agree with the PG expression feature and the transcriptomic analysis (Fig. 4). However, the results calculated by NADH and ADK are like the expression features of these genes and the transcriptomic analysis (Fig. 4). Furthermore, NADH + ADK as the coreference gene is not consistent with NADH or ADK alone (Fig. 4). Taken together, either NADH or ADK are the most suitable reference genes for RT–qPCR analysis in VBT fruit development.

#### Differential expressed genes from transcriptomic analysis validation using NADH

**Figure 5:**
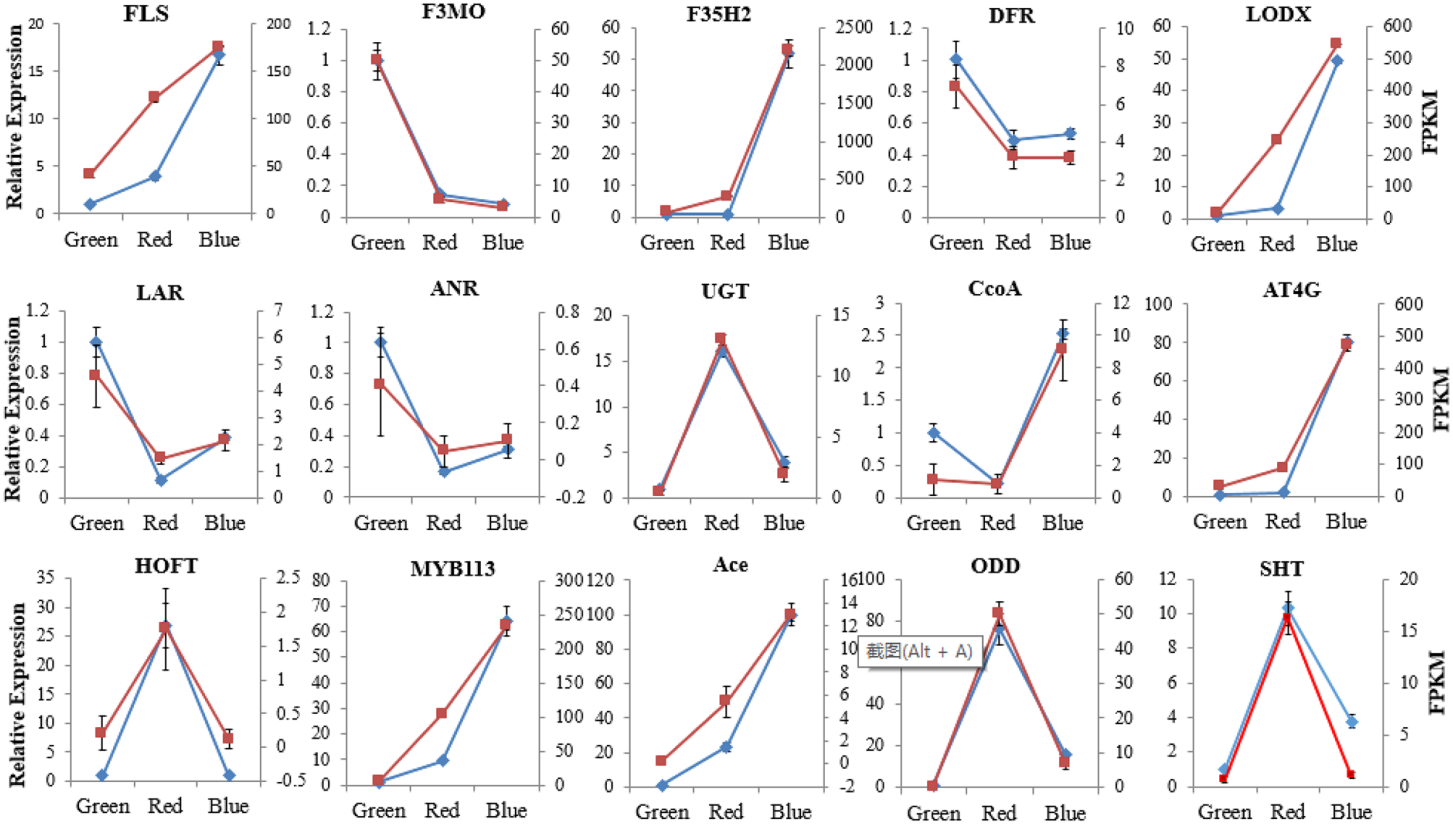
Validation of the DEGs in three ripening stage using NADH. FLS, flavonol synthase; F3MO, flavonoid 3′-monooxygenase; F35H2, flavonoid 3′,5′-hydroxylase 2; DFR, dihydroflavonol 4-reductase; LDOX, leucoanthocyanidin dioxygenase; LAR, leucoanthocyanidin reductase; ANR, anthocyanidin reductase; UGT, UDP-glycosyltransferase; CcoA, caffeoyl-CoA O-methyltransferase; At4g, probable caffeoyl-CoA O-methyltransferase; HOFT, hydroxypalmitate O-feruloyl transferase; MYB113, transcription factor MYB113; Ace, acetylajmalan esterase; ODD, 2-oxoglutarate-dependent dioxygenase; SHT, shikimate O-hydroxycinnamoyl-transferase. The blue line indicates RT-qPCR data normalized by NADH, and the red lines represent FPKM values. Both RT-qPCR experiments and RNA-seq data were repeated at least three times. Pictures were drawn in the Microsoft excel based the RT-qPCR data and FPKM value.

To further evaluate the reliability of the reference genes, the expression levels of 15 flavonoid synthesis-related genes were normalized to the expression level of NADH. These genes encode shikimate O-hydroxycinnamoyltransferase (SHT), flavonol synthase (FLS), flavonoid 3′-monooxygenase (F3MO), flavonoid 3′,5′-hydroxylase 2 (F35H2), dihydroflavonol 4-reductase (DFR), leucoanthocyanidin dioxygenase (LDOX), leucoanthocyanidin reductase (LAR), anthocyanidin reductase (ANR), UDP-glycosyltransferase 74B1 (UGT), caffeoyl-CoA O-methyltransferase (CcoA), probable caffeoyl-CoA O-methyltransferase (At4g), hydroxypalmitate O-feruloyl transferase (HOFT), transcription factor MYB113 (MYB113), acetylajmalan esterase (Ace), and 2-oxoglutarate-dependent dioxygenase (ODD) (Table S4). The results showed that the Pearson correlation coefficients of the 12 genes are above 0.9 (Fig. 5, Table S5), which indicates that their RT–qPCR results are highly consistent with the FPKM values in the transcriptome. In addition, the Pearson correlation coefficients of *FLS and LAR2*, and *ANR2* arranged from 0.81 to 0.89 (Table S5), indicating a lower correlation. However, their expression patterns were also highly consistent with the transcriptomic data. Taken together, the reference gene NADH is highly reliable for RT–qPCR analysis in the fruit ripening stage.

## Discussion

The VBT plant is well-known for producing “Wumifan” and is also used widely as an herbal medicine in China. However, the nutrients in its fruit have been ignored in previously reported studies. As a “wild-blueberry,” there are diverse and abundant amino acids, minerals, and unsaturated fatty acids in fruits, which are beneficial for human health^1^. Unlike blueberry, VBT plants are universally grown in the mountains in southern China, which suggests that this species is adapted the environment in China. Additionally, the fruit ripening stage is in autumn. Therefore, these features make VBT definitively specific to the Ericaceous family of plants. Blueberry are always ripened in early summer, when the rainy season has come, which dramatically impacts their production. Discovery of the genes controlling the fruit ripen time of VBT would allow their use in blueberry breeding, which would considerably benefit blueberry cultivation and production.

To comprehensively explore the VBT genome, we sequenced its genome and transcriptome. We found that no standard reference gene is suitable for RT–qPCR analysis. Reference genes, such as actin, tubulin, tef1, NADP, and ubiquitin, are widely used in different species^18,20,22–24^. However, appropriate reference genes are not uniform across diverse species, and even in different treatments or tissues of the same species^18–23^. We also attempted to use *actin* as the reference gene for validation of the expression levels according to the transcriptomic data. However, the RT–qPCR results are not stable, especially the Ct values, which are dramatically varied in various tissues. Therefore, reference screening is necessary for RT–qPCR analysis in VBT. To screen for the best reference genes in analysing the genes involved in fruit ripening of VBT, we obtained ten genes that had similar FPKM values in various ripening stages. The genes were analysed using GeNorm, NormFinder, BestKeeper. Thus, because the scores of a former reference gene that was evaluated by the three methods are not uniform, many researchers then selected genes with high scores in GeNorm and NormFinder, or selected genes using a combination of the three methods^18,20,21,23^. As a result, the best reference gene remained to be identified.

VBT is a unique species in China. Because of its high medicinal value and rich nutrient qualities, the molecular mechanisms involved in nutrient and active biochemical component synthesis in VBT should be better understood^1,2,15,28^. Our group sequenced the genome and transcriptome of VBT. However, the validation of the differentially expressed genes in the transcriptome involved in different fruit˗development stages using the *actin* gene as a normalizer was not stable^23^, and the results showed great differences in the same samples. The best reference gene in studying the development of fruits is not *actin*, which implies that it is not adaptable for validation of the genes involved in fruit development. Therefore, we obtained ten candidate reference genes from the transcriptomic data, that have similar gene expression levels in different fruit development stages. Furthermore, they wereevaluated by GeNorm, NormFinder, BestKeeper and a comprehensive ranking. The analysis showed that the comprehensive ranking values of several genes were remarkably close, suggesting that actin2, ADK, actin1 and NADH can be used as reference genes for gene expression analysis (Table 2). However, the Ct values for the *actin1* gene were too high in various ripen fruit stages of VBT (Table 1), which should not be beneficial for further analysis. Therefore, three genes, actin2, ADK and NADH, were selected for further analysis.

Ethylene is a key chemical involves in fruit development. During VBT fruitripening ethylene is continuously synthetized, accumulated, and released to the environment. To synthetize ethylene, the marker gene ACOs is dramatically expressed at the early stages of ripening, at the red fruit stage in blueberry, which means that these genes could be used to evaluate the reliability of reference genes. Therefore, we evaluated the expression levels of ACO genes using ADK, NADH, and NADH+ADK as reference genes. As a result, we found that both ADK and NADH are dependable for RT–qPCR analysis. Furthermore, the relative transcript levels of 15 DEGs predicted by transcriptomic analysis were also normalized to NADH based on RT–qPCR data. Pearson correlation coefficients between transcriptomic data and RT–qPCR data of the 12 genes normalized by the expression level of the NADH gene were above 0.9 in VBT fruit, which demonstrates the high reliability of the reference genes for characterizing gene expression levels in the fruit ripening stage.

## Conclusion

VBT plants have great value for producing “Wumifan,” enhancing herbal medicine, and advancing blueberry breeding. To explore its molecular content, further studies on the VBT genome and proteome should be conducted. RT–qPCR is a major method for evaluating gene expression levels. However, no candidate reference genes had been comprehensively screened in VBT fruits. In this study, we assessed ten reference genes and found that actin2, NADH and ADK genes are the most reliable reference genes for RT–qPCR analysis across all stages of VBT fruit ripening. Furthermore, the expression levels of 3 maker genes and 15 candidate differentially expressed genes in three fruit ripening stage were evaluated using NADH or ADK, both showed high consistence with the FPKM values in the transcriptome. These results will benefit further studies on the function of VBT genes involved in fruit ripening, fruit nutrient and bioactive substance production.

## Supplementary Information

Supplementary Information 1.

Supplementary Information 2.

Supplementary Information 3.

Supplementary Information 4.

Supplementary Information 5.

## Data Availability

Transcriptomic data are available in the NCBI Sequence Read Archive (SRA) database (Accession Numbers: BioProject PRJNA794927).
